# Provider Decisions to Treat Respiratory Illnesses with Antibiotics: Insights from a Randomized Controlled Trial

**DOI:** 10.1371/journal.pone.0152986

**Published:** 2016-04-04

**Authors:** Angela R. Branche, Edward E. Walsh, Nagesh Jadhav, Rachel Karmally, Andrea Baran, Derick R. Peterson, Ann R. Falsey

**Affiliations:** 1 Department of Medicine, University of Rochester, Rochester, NY, United States of America; 2 Department of Medicine, Rochester General Hospital, Rochester, NY, United States of America; 3 Department of Biostatistics and Computational Biology, University of Rochester, Rochester, NY, United States of America; Université Paris Descartes; AP-HP, Groupe Hospitalier Cochin-Saint-Vincent-de-Paul, FRANCE

## Abstract

**Rationale:**

Lower respiratory tract illness (LRTI) frequently causes adult hospitalization and antibiotic overuse. Procalcitonin (PCT) treatment algorithms have been used successfully in Europe to safely reduce antibiotic use for LRTI but have not been adopted in the United States. We recently performed a feasibility study for a randomized clinical trial (RCT) of PCT and viral testing to guide therapy for non-pneumonic LRTI.

**Objective:**

The primary objective of the current study was to understand factors influencing PCT algorithm adherence during the RCT and evaluate factors influencing provider antibiotic prescribing practices for LRTI.

**Study Design:**

From October 2013-April 2014, 300 patients hospitalized at a community teaching hospital with non-pneumonic LRTI were randomized to standard or PCT-guided care with viral PCR testing. Algorithm adherence data was collected and multivariate stepwise logistic regression of clinical variables used to model algorithm compliance. 134 providers were surveyed anonymously before and after the trial to assess knowledge of biomarkers and viral testing and antibiotic prescribing practices.

**Results:**

Diagnosis of pneumonia on admission was the only variable significantly associated with non-adherence [7% (adherence) vs. 26% (nonadherence), p = 0.01]. Surveys confirmed possible infiltrate on chest radiograph as important for provider decisions, as were severity of illness, positive sputum culture, abnormal CBC and fever. However, age, patient expectations and medical-legal concerns were also at least somewhat important to prescribing practices. Physician agreement with the importance of viral and PCT testing increased from 42% to 64% (p = 0.007) and 49% to 74% (p = 0.001), respectively, after the study.

**Conclusions:**

Optimal algorithm adherence will be important for definitive PCT intervention trials in the US to determine if PCT guided algorithms result in better outcomes than reliance on traditional clinical variables. Factors influencing treatment decisions such as patient age, presence of fever, patient expectations and medical legal concerns may be amenable to education to improve PCT algorithm compliance for LRTI.

## Introduction

Acute respiratory infections are a frequent cause of medically attended illness in older adults and often require hospitalization. The management of mild acute respiratory infection in the outpatient setting is clear, as most infections are due to viruses, illnesses are self-limited, and data showing the safety and benefits of withholding antibiotics are robust.[1–4] Interventions to reduce unnecessary antibiotics can be focused on provider education and strategies to change behavior.[5–11] However, physicians who provide care for patients hospitalized with lower respiratory tract infections (LRTI) are faced with more challenging decisions. Data regarding the etiology of respiratory illnesses in hospitalized adults are incomplete and current microbial diagnostic tests frequently do not allow clinicians to rule out bacterial infection with certainty.[12–14] Highlighting the problem further, we recently reported that 39% of adults hospitalized with viral infections had evidence of bacterial co-infection.[15] Thus, it is not unreasonable when faced with diagnostic uncertainty to treat with antibiotics. Professional societies have espoused an approach de-emphasizing diagnostic testing and promoting empiric antibiotic treatment for community acquired pneumonia (CAP).[12, 16] In addition, the Center for Medicaid Services (CMS) has added pressure to quickly administer antibiotics as a quality measure for patients with pneumonia leading to treatment of many patients with “possible pneumonia” that actually have other conditions.[17–19] The wisdom of these approaches in an era of rising rates of *Clostridium difficile* colitis and multi-drug resistant organisms is now being questioned.[20–21] The use of biomarkers and definitive viral diagnostic testing may provide more accurate targeting of antibiotics to those most likely to benefit, and minimize adverse outcomes.[22–24]

Procalcitonin (PCT) is a peptide precursor of calcitonin released by parenchymal cells in the presence of bacterial toxins which has been shown to be strongly associated with bacterial infections. Conversely, in patients with viral infection, PCT release is down-regulated resulting in markedly low serum levels. It was first used in patients with suspected bacterial infections or sepsis to differentiate between infectious and noninfectious causes of systemic inflammatory response syndrome.[25–30] Despite conflicting results on the clinical utility of PCT to reduce antibiotic exposure or predict mortality, it has been adopted into widespread practice in critical care settings and is currently approved by the United States Food and Drug Administration (FDA) for use in patients with suspected sepsis.[27]

Recent reports from Europe suggest that serum procalcitonin (PCT) may be effectively used to safely reduce antibiotic use in LRTI by helping to differentiate between bacterial and viral respiratory illnesses and guide duration of therapy.[31,32] A large multicenter trial, the *ProHOSP* study, randomized patients with both pneumonic and non-pneumonic LRTI to PCT guided care or standard care and demonstrated that even in patients at highest risk for complicated bacterial infections, a PCT treatment algorithm can be used to shorten antibiotic duration without harm to patients.[24] However, the FDA has not approved this use of PCT in the United States and implementing this strategy in US institutions will be impeded by both current US medical society guidelines and lack of physician experience with PCT treatment algorithms.[33–35] The latter issue is supported by Europeans reports of increased algorithm adherence in PCT experienced centers compared to PCT naïve centers.[30, 33] Thus, acceptance of PCT treatment algorithms in the US will likely be an iterative process requiring larger US based clinical trials and a more measured first step assessing safety and utility in low risk patients.

We recently conducted a randomized clinical trial assessing the feasibility of combining a PCT based algorithm with rapid viral testing to guide antibiotics in patients hospitalized with non-pneumonic LRTI.[36] Physician adherence to the algorithm was 64% during the trial, and although there were no significant differences in overall antibiotic use between intervention and standard care patients, subgroup analyses revealed encouraging results with significantly fewer viral positive and low PCT subjects discharged on antibiotics. Importantly, antibiotic duration was significantly shorter in algorithm adherent versus nonintervention patients. Since optimizing adherence to treatment algorithms will be important to the success of future larger trials, we analyzed clinical and laboratory factors associated with algorithm adherence during the trial and compared these results to a physician survey of knowledge and attitudes regarding viral testing, biomarkers and antibiotic prescribing practices for respiratory illness.

## Methods

### Randomized Clinical Trial (NCT01907659)

The methods and results of this trial have been previously described in detail.[34] Briefly, 300 patients hospitalized with non-pneumonic LRTI were randomized equally to standard care or PCT guided care in combination with multiplex viral PCR testing, at a large community teaching hospital. Subjects or their healthcare representative provided written informed consent and the study was approved by the RGH and University of Rochester Institutional Review Boards. The majority of patients were cared for by physicians in the division of hospital medicine. Prior to the study, providers were formally educated regarding the causes of respiratory infections, current antibiotic treatment guidelines and antibiotic complications as well as the use of PCT algorithms to guide antibiotic therapy. Adults with symptoms compatible with LRTI were identified daily and those deemed high risk for serious bacterial infection were excluded (ICU requirement, active chemotherapy or radiation, immunosuppression, hypotension, ≥ 15% band forms in peripheral blood, definitive infiltrate on chest radiograph [CXR]). Patients with a clinical admission diagnosis of pneumonia and ambiguous radiographic readings such as “possible infiltrate” were included if the aforementioned exclusion criteria were not present. Nose and throat swabs for PCR and two serum samples drawn at least 12 hours apart for PCT testing were collected.

Subjects randomized to intervention had PCT and multiplex viral testing (FilmArray, Biofire Diagnostics) performed immediately. Multiplex viral testing included: influenza A & B (FLU), respiratory syncytial virus (RSV), parainfluenza viruses 1–4 (PIV), rhinovirus (HRV), adenovirus, human metapneumovirus (HMPV), 4 human coronaviruses (HCoV) and 3 atypical bacteria (*Mycoplasma pneumoniae*, *Bordetella pertussis and Chlamydia pneumoniae)*.

Results were available in the electronic medical record 2–3 hours after enrollment and the treating team notified by text page and simultaneous email providing the PCT algorithm interpretation. The PCT algorithm recommendations were as follows:

≤ 0.1 ng/ml: antibiotics strongly discouraged
0.11 to 0.24 ng/ml: antibiotics discouraged
0.25 to 0.49 ng/ml: antibiotics encouraged
≥ 0.5 ng/ml: antibiotics strongly encouraged.

Subjects in the standard care group had routine testing according to institutional guidelines performed at the discretion of the provider. Routine molecular testing for influenza and RSV was available for all patients. Bacterial blood cultures were obtained at the discretion of the providing team and results were recorded for subjects in both arms. Results of bacterial sputum cultures were accepted only from adequate samples, defined by standard microbiologic criteria (i.e., gram stain with <10 epithelial cells and >25 polymorphonuclear cells per high powered field [HPF]) [37]

Antibiotic exposure was measured as days of antibiotic therapy, discontinuation of antibiotics within 48 hours, and discharge on antibiotics. Algorithm adherence was defined as antibiotics discontinued ≤72 hours of admission for intervention subjects with PCT values ≤ 0.24 ng/ml or continuation of antibiotics for >72 hours in subjects with PCT values of >0.25 ng/ml.

### Provider Surveys

Three surveys to assess provider knowledge and beliefs about respiratory infections, viral testing and serum biomarkers were conducted. Each survey was provided via email link to internal medicine resident physicians, attending physicians and midlevel providers and results anonymously returned.

The first survey was conducted prior to informational sessions and the clinical trial, and consisted of 7 questions concerning the percentage of respiratory illnesses believed due to bacteria, viruses or mixed bacterial viral infections, attitudes regarding the value of viral and PCT testing, and perception of antibiotic overuse for respiratory illness. A similar survey was conducted one month after completion of the trial with three additional questions to determine if providers understood PCT threshold values used in the treatment algorithm and believed that PCT and viral testing results influenced their management decisions during the trial.

A final survey was administered 1 year after the trial concluded to further evaluate provider knowledge about specific viral infections and to assess in greater detail those factors that might influence algorithm non-adherence. The survey questions related to a hypothetical scenario of a stable immunocompetent patient without definitive pneumonia on CXR with a low PCT value.

### Statistical Analysis

Categorical variables were summarized by counts and proportions, and compared using Fisher’s exact test. Medians and interquartile ranges (IQR) were used to describe continuous variables, with comparisons based on the nonparametric Wilcoxon rank-sum test for two independent variables and the Kruskal-Wallis test for significance of the difference among more than two distributions. For intervention patients with PCT ≤ 0.24ng/ml, stepwise logistic regression was used to model algorithm compliance as a function of a subset of clinical covariates including: age, log(length of symptoms prior to admission), sputum culture results, signs and symptoms of illness, admission diagnosis, CXR results, log(WBC), log(PCT) and viral testing. Positively skewed continuous variables were log transformed to reduce skewness and mitigate the effects of potential outliers, since unlike the Wilcoxon and Kruskal-Wallis tests, logistic regression is not robust to outliers. All tests were performed at the 2-sided 0.05 level of significance.

Seven subjects discharged on chronic antibiotic therapy were excluded from analyses assessing antibiotic exposure and algorithm compliance. Four subjects in the nonintervention did not have a PCT value and 3 subjects in the intervention arm did not have a chest x ray.

## Results

### RCT: Summary Results

**Fig 1:**
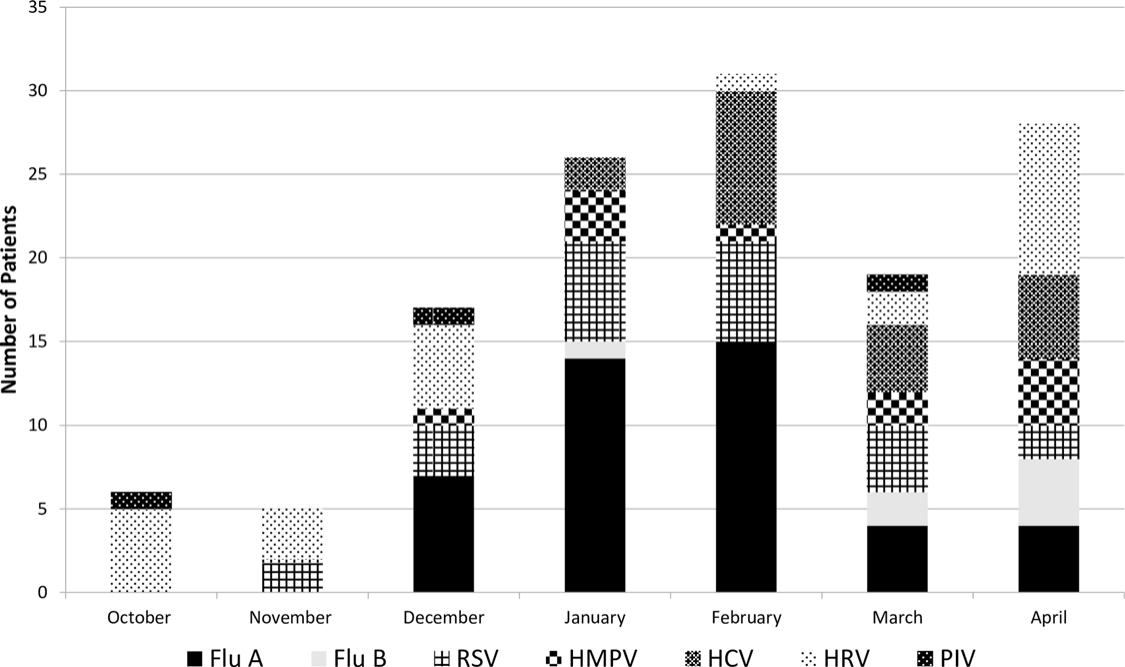
Distribution of specific viral infections during the study period (October 2013- April 2014). Each specific virus is identified by a different shade or pattern and the total numbers are distributed by month. The total numbers per month represent viral diagnosis made in both intervention and nonintervention subjects, though only the diagnoses of intervention subjects were revealed to treating providers during the trial.

From October 2013 to April 2014, 151 eligible patients were randomized to intervention and 149 to nonintervention.[36] The most common admission diagnoses were acute exacerbation of chronic obstructive pulmonary disease (AECOPD) (39%), asthma exacerbation (20%), pneumonia (19%), congestive heart failure (CHF) (8%) and influenza infection (7%). Viruses were identified in 42% (Fig 1) and bacterial diagnoses were made in 9–10% of patients, most by sputum culture. The majority of intervention patients (80%) had low admission PCT values (≤0.24 ng/ml) as did the majority of subjects with a viral diagnosis. There were no significant differences in measures of antibiotic exposure or the number of adverse events between intervention and nonintervention subjects. Importantly, antibiotic duration was significantly shorter in algorithm adherent intervention patients vs. nonintervention patients (2.0 vs.4.0 days, p = 0.004). In addition, subgroup analysis demonstrated fewer subjects with low PCT and any positive viral test were discharged on antibiotics (20% vs.45%, p = 0.002).

### RCT: Factors Associated with Algorithm Adherence

Overall algorithm adherence was 64% in the clinical trial. Provider adherence to the algorithm was somewhat greater (77% vs. 60%) when PCT values were high (PCT ≥ 0.25 ng/ml, i.e., antibiotics recommended), but this difference was not statistically significant (p = 0.14). (Table 1) Antibiotics were not given or were discontinued contrary to algorithm recommendations in 7 high PCT subjects (median PCT 1.50 ng/ml [range 0.35–4.56]) without adverse outcomes. Interestingly, five were viral positive (2 Flu A, 1 Flu B and 2 HRV). One high PCT patient whose admission diagnosis was COPD exacerbation was never started on antibiotics and discharged with a diagnosis of acute bone injury three days later. The final high PCT subject who received ≤ 3 days of antibiotics was found to have acute renal failure on admission and had a prolonged hospital stay complicated by gastrointestinal hemorrhage.

**Table 1:** Provider decision to follow or overrule the PCT treatment algorithm stratified by PCT and viral testing results.

	Low PCT (≤ 0.24 ng/ml)				High PCT (> 0.25 ng/ml)			
	Viral Positive N = 49	Viral Negative N = 69	P Value	Total N = 118	Viral Positive N = 15	Viral negative N = 15	P Value	Total N = 30
**Algorithm Followed, No. (%)**	34 (69)	37 (54)	0.09	71 (60)^a^	10 (67)	13 (87)	0.39	23 (77)
**Algorithm Overruled No. (%)**	15 (31)	32 (46)		47 (40)	5 (33)	2 (13)		7 (23)

^a^3 subjects excluded from analysis due to chronic antibiotic use.

In contrast, only 60% of providers followed algorithm recommendations to discontinue antibiotics in subjects with a low PCT value. Univariate analysis of algorithm adherence in subjects with low PCT values revealed trends toward increased adherence for patients with a viral diagnosis or asthma, lower WBC, and decreased adherence for those with a positive adequate bacterial sputum culture or a chest radiograph read as “possible infiltrate. (Table 2) A clinical admission diagnosis of pneumonia was significantly associated with overruling the algorithm and in multivariate stepwise logistic regression analysis, was the only variable significantly associated with algorithm compliance in low PCT subjects. (OR = 0.22; 95% CI 0.07, 0.68, p = 0.01). Of 12 subjects with an admission diagnosis of pneumonia for whom the algorithm was overruled, six had microbiological diagnoses made which included 3 viral infections without evidence of bacterial infection (2 influenza and 1 RSV) and 3 bacterial infections (1 *Mycoplasma* pneumoniae, and two with positive bacterial sputum cultures (*Haemophilus influenza* and *Moraxella catarrhalis*).

**Table 2:** Factors affecting provider adherence to algorithm in subjects with low PCT value.

	Algorithm Followed N = 71^a^	Algorithm Overruled N = 47	P value
**Age, median (IQR)**	58.0 (16.0)	60.0 (17.0)	0.37
**Length of symptoms prior to admission, median (IQR)**	4.0 (11.0)	3.0 (4.0)	0.68
**Diagnostic Tests**			
**PCT, median (IQR)**	0.05 (0)	0.05 (0.02)	0.40
**WBC, median (IQR)**	8.3 (4.1)	9.8 (4.5)	0.08
**Virus positive, No. (%)**	34 (48)	15 (32)	0.09
**Positive adequate sputum culture** ^b^**, No. (%)**	2 (3)	6 (13)	0.06
**Possible infiltrate on CXR, No. (%)**	13 (19)	16 (34)	0.08
**Symptoms, No (%)**			
**Cough**	64 (90)	44 (94)	0.74
**Sputum**	51 (72)	40 (85)	0.12
**Fever**	12 (17)	6 (13)	0.61
**Rales**	16 (23)	16 (34)	0.21
**Wheezing**	54 (76)	28 (60)	0.07
**CURB 65 Score, median (IQR)**	1.0 (2.0)	1.0 (2.0)	0.35
**Clinical Admission Diagnosis, No. (%)**			
**COPD**	29 (41)	17 (36)	0.70
**Pneumonia**	5 (7)	12 (26)	0.01
**Asthma**	23 (32)	8 (17)	0.09

^a^ 3 subjects excluded from analysis due to chronic antibiotic use.

^b^ Adequate samples were defined by standard microbiologic criteria (i.e., gram stain with <10 epithelial cells and >25 polymorphonuclear cells per high powered field [HPF]).

### Pre-Study and Post-Study Provider Surveys

Surveys were sent to the 134 providers in the department of medicine (58 resident physicians, 24 midlevel providers and 53 attending physicians), the majority of whom participated in the care of study patients in both arms of the RCT. Ninety-five providers responded to the pre-trial survey and 70 to the post-trial survey. Survey results indicated no significant change after the trial in provider perception regarding the frequency that respiratory infections are due to bacteria, viruses or mixed viral bacterial pathogens. The majority of responders indicated that 41–75% of respiratory illnesses were due to viral infections both pre and post-study (86% vs. 93%, p = 0.33). In addition, the majority of providers in the pre and post study surveys felt that bacterial infections, including mixed viral-bacterial infections, occurred in ≤40% of patients admitted with LRTI.

In contrast, provider opinion regarding the utility of viral and PCT testing changed significantly in the post-trial survey (Table 3). Physician agreement with the statement that “viral testing was important for patient care” increased from 42% to 64% after the study (p = 0.007), and the percentage of providers who felt that PCT testing was important for patient care increased from 49% to 74% after the study (p = 0.001). The majority of providers felt the combination of viral and biomarker testing was more useful than either test alone, with provider agreement increasing from 62% to 81% after the trial (p = 0.009). Nearly all providers felt that antibiotics are overused in patients hospitalized with LRTI and by the end of the trial 100% agreed with this statement. Finally, when asked how frequently results of PCT and viral testing influenced their decision to continue, stop or start antibiotics, most providers responded that results either frequently (22%) or sometimes (68%) influenced antibiotic prescribing practices.

**Table 3:** Comparison of physician agreement (agree or strongly agree) in the pre and post study surveys with the following statements.

	Pre-Study (N = 95)	Post-Study (N = 70)	P value
**Viral testing is important for patient care**	40 (42)	45 (64)	0.007
**PCT testing is important for patient care**	47 (49)	52 (74)	0.001
**Combination testing is better than either alone**	59 (62)	57 (81)	0.009
**Antibiotics are overused in patients hospitalized with LRTI**	92 (97)	70 (100)	0.26

### Provider Survey at 12 Months

A follow-up survey was conducted one year after trial completion to assess the long term impact of the clinical trial on physician knowledge and attitudes regarding the utility of PCT and viral testing. Respondents included 42 attending physicians, 39 resident physicians and 13 midlevel providers. Of the 94 providers surveyed, 72% were familiar with the serum biomarker PCT and 76% correctly identified the algorithm recommendation for a PCT value < 0.24 ng/ml (i.e., antibiotics not recommended). (Table 4) Most providers indicated a desire that PCT testing be routinely available in the hospital (82% of resident physicians and 76% of attending physicians).

**Table 4:** 12 month post-hoc survey assessing physician perceptions regarding PCT.

94 Respondents	Yes	No	Unsure	No Response
**Are you familiar with the serum biomarker PCT?**	68 (72)	26 (28)	0	0
**Are antibiotics recommended for PCT < 0.24 ug/nl?**	1 (1)	71 (76)	22 (23)	6
**Would you ever be willing to stop ABX in patients with clinical pneumonia?**	33 (35)	40 (43)	21 (22)	5
**Would want PCT routinely available in the hospital?**	71 (76)	4 (4)	19 (20)	6

We also sought to determine if familiarity with various respiratory viral infections influenced algorithm adherence. Providers were universally familiar with influenza (100%) and RSV (99%), and less so with coronaviruses (66%) and HMPV (19%). (Table 5) These results did not differ by years in practice. Providers were also asked to assess which viruses were associated with pneumonia or severe disease and while many agreed that influenza (97%) and RSV (92%) could lead to severe illness, significantly fewer providers felt that HMPV (23%) or HCoV (45%) could do so. Although most were familiar with HRV (82%), few felt this virus might cause severe illness (27%). Nevertheless, algorithm adherence was similar regardless of the specific viral diagnosis and was somewhat greater than those without a viral diagnosis (influenza & RSV [71% adherence] vs. other viruses [68% adherence] vs. no virus [54% adherence], p = 0.09).

**Table 5:** 12 month post-hoc survey assessing provider familiarity with 5 common respiratory viruses.

94 Respondents	HMPV	HCoV	RSV	PIV	Influenza	HRV
**Which of the follow viruses are you familiar with?**	18 (19)	61 (66)	93 (99)	82 (87	94 (100)	77 (82)
**Which of the following viruses do you believe cause severe illness or pneumonia?**	21 (23)	42 (45)	86 (92)	58 (62)	90 (97)	25 (27)

**Fig 2:**
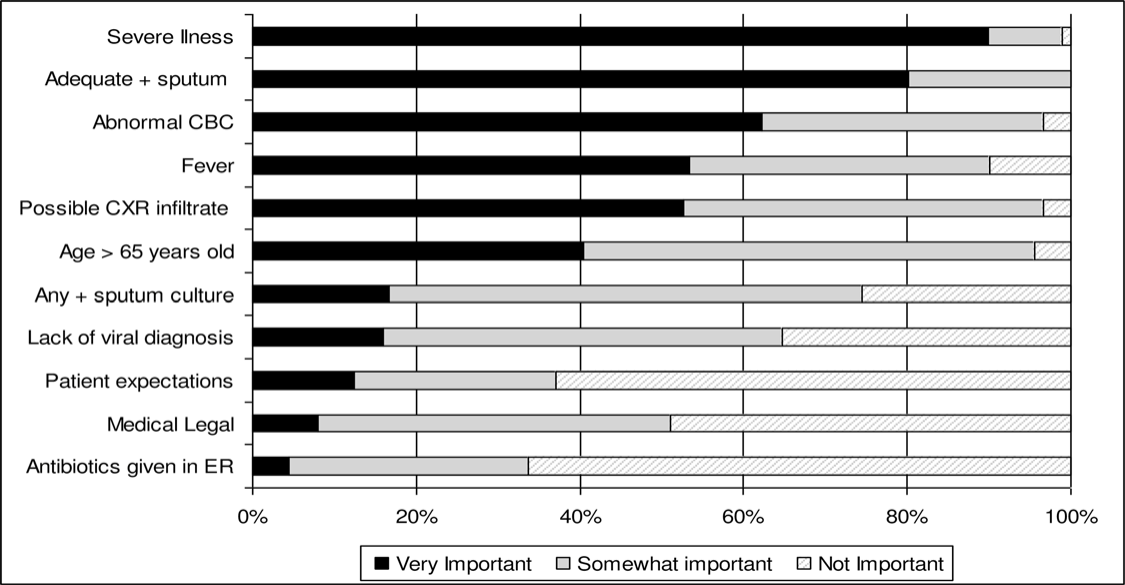
Provider survey response of the influence of eleven clinical factors on treatment decisions. The survey included a scenario of a stable immunocompetent patient without definitive pneumonia on CXR with a low PCT value. Providers were queried as to factors that might lead to a decision to disregard the algorithm and prescribe antibiotics.

Next, we asked providers to rate 11 clinical factors as either “very important”, “somewhat important” or “not important” in deciding to continue antibiotics in a scenario describing a stable, non-immunocompromised patient with a respiratory illness and a low PCT value despite algorithm recommendations to not give antibiotics. The most important factors were severity of illness and a positive adequate bacterial sputum culture in the decision to treat with antibiotics (Fig 2). Although the data from the clinical trial indicated that a clinical diagnosis of pneumonia was a significant factor associated with algorithm non-adherence, only 53% of respondents considered a “possible infiltrate on CXR” to be “very important”. However, 97% considered an ambiguous CXR at least “somewhat important” in the decision to continue antibiotics highlighting the relevance of ambiguity when diagnosing radiographic pneumonia. Patient expectations, medical legal issues, and antibiotics started in the emergency department were infrequently deemed as “very important”, however, half considered medical legal concerns at least “somewhat important”. There were also a few significant differences between resident and attending physician responses. Seventy-three percent of residents felt that the presence of fever was “very important” for their decision to give or withhold antibiotics, while 33% of attending physicians rated fever as “very important” (p = 0.002). The lack of a viral diagnosis (30% vs. 5%, p = 0.005) and age over 65 years (54% vs. 29% p = 0.02) were more important factors to residents compared to attending physicians.

Finally, the majority of survey participants felt that definitive randomized clinical trials using PCT algorithms (72%), better bacterial diagnostics (63%) or society guidelines (56%) would be “very important” in guiding best clinical practices for the treatment of respiratory infections in hospitalized patients. Better biomarkers were ranked “very important” least frequently at 43%.

## Discussion

Lower respiratory tract illness (LRTI) is a frequent cause of adult hospitalization and many of these infections are due to viruses.[38–40] Our pre and post-trial surveys indicate that the majority of providers have a good understanding of the relative percentage of respiratory infections caused by viruses, bacteria and mixed viral-bacterial infections. Yet antibiotic use remains high, despite increased availability of rapid viral diagnostics in our study population similar to the published literature.[41–43]

Although overall PCT algorithm compliance in our RCT was only 64%, data suggested that providers might be more likely to discontinue therapy early in patients with low PCT and a definitive viral diagnosis, although these differences were not statistically significant.[36] Interestingly, when providers did not continue antibiotics in subjects with high PCT values, 70% had viral diagnoses, again suggesting that identification of an alternative definitive pathogen might have influenced decision making. Consistent with this, our post-study survey found that the majority of physicians felt that the combination of PCT and viral tests was more helpful than either test alone. Taken together these findings suggest that clinicians like to know what a patient “has” (i.e., a virus) not just what they “don’t have” (i.e., a low PCT indicative of non-bacterial infection) when making antibiotic treatment decisions. Further education regarding the clinical features associated with illnesses caused by respiratory viruses, and specifically that viruses can cause clinical and radiographic pneumonia, may be helpful in increasing provider confidence in discontinuing antibiotics for patients with a known viral diagnosis.

Despite the encouraging results from Europe, larger PCT guided treatment trials for respiratory infections are needed in the US before it will be adopted into clinical practice. However, compliance with treatment algorithms, especially in patients with low PCT values, will be important if such trials are to be definitive in proving the value and safety of using PCT algorithms as a means of reducing unnecessary antibiotic use. Analysis of our clinical trial data found that there was a trend towards algorithm non-adherence in subjects with higher WBC, a positive adequate sputum culture, a possible infiltrate on CXR, and an admission diagnosis of pneumonia (Table 2), with only the latter being statistically significant. Consistent with these results, our survey identified positive adequate bacterial sputum cultures, abnormal peripheral blood counts, possible infiltrate on chest radiograph, as well as age and severity of illness as factors frequently considered by providers as “very or somewhat important” in deciding to override algorithm recommendations. While each of the above appear to be a reasonable factor when assessing patients for the likelihood of bacterial infection, many studies have shown that such clinical variables lack sensitivity and specificity in predicting bacterial infection.[44–47] Finally, though given less weight by providers, patient expectations, medical legal issues and antibiotics started in the emergency room were all at least “somewhat important” considerations in decision making. Many of these issues should be at least partially amenable to educational initiatives that among other things, remind physicians of the limited clinical impact of a single dose of antibiotics given in the ER compared to other treatment measures such as hydration, oxygen and bronchodilators and their culpability in giving antibiotics to very low risk patients who subsequently develop serious antibiotic related complications.

There are several limitations in the interpretation of our results. First, because the pre and post study surveys were conducted anonymously in order to elicit unbiased responses, we could not perform a paired-analysis. Thus, we could not fully assess changes in individual perceptions or account for selection bias. Secondly, the post-study surveys were not timed to optimally capture reasons for non-adherence to the PCT algorithm. Ideally, the information should have been captured immediately when providers made the decision to accept or overrule the PCT algorithm for a specific patient. Lastly, the sample size of our study is relatively small and the study was carried out in a single center, and thus may not be directly transferable to other clinical centers.

It is important to note that strict adherence to PCT guided algorithms should not be construed as best care in all cases, any more than current therapy guidelines should be. However when coupled with good clinical judgment, such guidelines and algorithms may improve overall outcomes. In future trials it will be important to prospectively monitor a more thorough assessment of the reasons why a physician might choose to overrule algorithm recommendations.

In conclusion, the development of strategies to reduce unnecessary antibiotic use can be an important supplement to existing hospital antibiotic stewardship programs.[30, 33] In the future, improved bacterial diagnostics such as transcriptional profiling of immune responses may fill this role.[40, 48, 49] In the interim, viral testing with PCT based algorithms may be of value. Our analysis suggests that addition of viral testing to a PCT algorithm is useful, and we have identified a number of issues that may be amenable to education. Our results should assist in design of definitive US randomized clinical trials of PCT guided algorithms, as these will be most influential in clinician acceptance of biomarker based approaches for the management of respiratory infections in adults.

## Supporting Information

S1 Appendix Pre-Trial Survey. (DOC)

S2 Appendix Post-Trial Survey. (DOC)

S3 Appendix 12 Month Follow-up Survey. (DOCX)

S4 Appendix Randomized Control Trial Report. Serum Procalcitonin Measurement and Viral Testing to Guide Antibiotic Use for Respiratory Infections in Hospitalized Adults: A Randomized Controlled Trial. Published in *Journal of Infectious Diseases* 2015.
(PDF)
