# Supplementary material for: Provider Decisions to Treat Respiratory Illnesses with Antibiotics: Insights from a Randomized Controlled Trial
Source: PLoS One. 2016 Apr 4;11(4):e0152986. doi: 10.1371/journal.pone.0152986 (PMC4820114; doi:10.1371/journal.pone.0152986)
Supplement: S1 Appendix — (DOC) [file pone.0152986.s001.doc]

**S1 Appendix. Pre-Trial Survey.**

1. How frequently do you believe respiratory infections are bacterial?

O <20% O 20-40% O 41-75% O >75%

2. How frequently do you believe respiratory infections are viral?

O <20% O 20-40% O 41-75% O >75%

3. What percent of viral infections are complicated by bacterial infections?

O <20% O 20-40% O 41-75% O >75%

4. Viral testing is important for management of respiratory infections.

O Strongly agree O Somewhat agree O Neutral O Somewhat disagree O Strongly disagree

5. Procalcitonin testing is important for management of respiratory infections.

O Strongly agree O Somewhat agree O Neutral O Somewhat disagree O Strongly disagree

6. The combination of viral and procalcitonin testing is more useful than either test alone.

O Strongly agree O Somewhat agree O Neutral O Somewhat disagree O Strongly disagree

7. Antibiotics are overused in patients hospitalized with respiratory infections.

O Strongly agree O Somewhat agree O Neutral O Somewhat disagree O Strongly disagree
