# Supplementary material for: Provider Decisions to Treat Respiratory Illnesses with Antibiotics: Insights from a Randomized Controlled Trial
Source: PLoS One. 2016 Apr 4;11(4):e0152986. doi: 10.1371/journal.pone.0152986 (PMC4820114; doi:10.1371/journal.pone.0152986)
Supplement: S3 Appendix — (DOCX) [file pone.0152986.s003.docx]

**S3 Appendix. 12 Month Follow-up Survey.**

Procalciton (PCT) is a serum biomarker that has been used to guide antibiotic therapy. Elevated levels are associated with sepsis. In Europe PCT algorithms have been used to guide antibiotic treatment of respiratory infections. Last year we performed a feasibility trial of PCT algorithms for respiratory infections at RGH and many of you participated. We would appreciate your thoughts regarding the utility of PCT and adherence to the algorithm.

We are interested specifically in patients hospitalized with respiratory illness you do **NOT** have definitive pneumonia on CXR, are stable (not in intensive care) and are not immunocompromised.

1. Are you familiar with PCT algorithms?

O Yes O No

2. Are antibiotics recommended for respiratory infection when levels are < 0.24ng/ml?

O Yes O No O Don’t know

3. Which of the following viruses are you familiar with? Check all that apply.

- Human Metapneumovirus
- Human Coronavirus
- Respiratory Syncytial Virus
- Parainfluenza virus
- Influenza
- Human Rhinovirus

4. Do you think any of the following viruses can cause severe illness or pneumonia? Check all that apply

- Human Metapneumovirus
- Human Coronavirus
- Respiratory Syncytial Virus
- Parainfluenza virus
- Influenza
- Human Rhinovirus

3. If a stable, non immunocompromised patient with respiratory illness has a low PCT what factors influence your decision to **continue** antibiotics?

O- Very important O-somewhat important O-not important

a. Lack of Viral Diagnosis

b. CXR- showing possible infiltrate per radiology report

c. Age greater than 65 years old

d. Presence of COPD or other lung disease

e. Positive adequate sputum culture

f. Positive poor quality sputum culture

g. Abnormal CBC ( high WBC or toxic granulation, dohle bodies)

h. Severe illness

i. Fever

j. Patient expectations

k. Colleague (ED or admitting MD) told patient they needed antibiotics

l. Medical-legal concerns

4. Would you ever be willing to stop or withhold antibiotics in a patient with a diagnosis of clinical pneumonia?

O Yes O No O Don’t know

5. What information would be most helpful if you were to consider not using antibiotics in some patients hospitalized with respiratory infections?

O- Very important O-somewhat important O-not important

a. Definitive randomized clinical trial published in a major journal

b. Society Guidelines

c. Better biomarkers

d. Better bacterial diagnostics

6. Would you like to see PCT available as a routine test at Rochester General Hospital?

O Yes O No O Don’t know or neutral
