# Supplementary material for: Provider Decisions to Treat Respiratory Illnesses with Antibiotics: Insights from a Randomized Controlled Trial
Source: PLoS One. 2016 Apr 4;11(4):e0152986. doi: 10.1371/journal.pone.0152986 (PMC4820114; doi:10.1371/journal.pone.0152986)
Supplement: S4 Appendix — Serum Procalcitonin Measurement and Viral Testing to Guide Antibiotic Use for Respiratory Infections in Hospitalized Adults: A Randomized Controlled Trial. Published in Journal of Infectious Diseases 2015. (PDF) [file pone.0152986.s004.pdf]

Journal of Infectious Diseases Advance Access published June 1, 2015

MAJOR ARTICLE

Serum Procalcitonin Measurement and Viral Testing to Guide Antibiotic Use for Respiratory Infections in Hospitalized Adults: A Randomized Controlled Trial

Angela R. Branche,[1] Edward E. Walsh,[1,3] Roberto Vargas,[4] Barbara Hulbert,[4] Maria A. Formica,[3] Andrea Baran,[2] Derick R. Peterson,[2] and Ann R. Falsey[1,3]

[1]Department of Medicine, and [2]Department of Biostatistics and Computational Biology, University of Rochester, [3]Department of Medicine, and [4]Department of Laboratory Sciences, Rochester General Hospital, New York

Background. Viral lower respiratory tract illness (LRTI) frequently causes adult hospitalization and is linked to antibiotic overuse. European studies suggest that the serum procalcitonin (PCT) level may be used to guide antibiotic therapy. We conducted a trial assessing the feasibility of using PCT algorithms with viral testing to guide antibiotic use in a US hospital.

Methods. Three hundred patients hospitalized with nonpneumonic LRTI during October 2013–April 2014 were randomly assigned at a ratio of 1:1 to receive standard care or PCT-guided care and viral PCR testing. The primary outcome was antibiotic exposure, and safety was assessed at 1 and 3 months.

Results. Among the 151 patients in the intervention group, viruses were identified in 42% (63), and 83% (126) had PCT values of <0.25 µg/mL. There were no significant differences in antibiotic use or adverse events between intervention patients and those in the nonintervention group. Subgroup analyses revealed fewer subjects with positive results of viral testing and low PCT values who were discharged receiving antibiotics (20% vs 45%; $P = .002$) and shorter antibiotic durations among algorithm-adherent intervention patients versus nonintervention patients (2.0 vs 4.0 days; $P = .004$). Compared with historical controls (from 2008–2011), antibiotic duration in nonintervention patients decreased by 2 days (6.0 vs 4.0 days; $P < .001$), suggesting a study effect.

Conclusions. Although antibiotic use was similar in the 2 arms, subgroup analyses of intervention patients suggest that physicians responded to viral and biomarker data. These data can inform the design of future US studies.

Clinical Trials Registration. NCT01907659.

Keywords. procalcitonin; viral testing; antibiotic use; respiratory infections.

Lower respiratory tract infection (LRTI) commonly causes adult hospitalization, and viruses account for many of these illnesses [1–4]. Increased availability of multiplex polymerase chain reaction (PCR) assays allows clinical laboratories to rapidly detect a wide variety of respiratory viruses [5, 6]. Despite viral detection, most patients in US hospitals receive broad-spectrum antibiotics, partly because of concerns about bacterial coinfection [7–9]. Reports from Europe suggest that elevated serum procalcitonin (PCT) levels predict bacterial infection and that PCT algorithms can be used to safely guide antibiotic use in LRTI, resulting in significant reductions in antibiotic duration [10, 11]. However, PCT-based treatment algorithms for respiratory infections have not been widely adopted in the United States [12–14]. In a prior study of adults hospitalized with respiratory illnesses, we showed that viral infection was common and that, of those with viruses, 60% had no evidence of bacterial infection, although most received antibiotics [15]. Results of a post-hoc physician survey

Received 10 March 2015; accepted 3 April 2015.
Presented in part: IDWeek, San Diego, California, 7–11 October 2014.
Correspondence: Angela R. Branche, MD, Infectious Disease Unit, University of Rochester, 601 Elmwood Ave, Rochester, NY 14642 (angela_branche@urmc.rochester.edu).

The Journal of Infectious Diseases®
© The Author 2015. Published by Oxford University Press on behalf of the Infectious Diseases Society of America. All rights reserved. For Permissions, please e-mail: journals.permissions@oup.com.
DOI: 10.1093/infdis/jiv252

Downloaded from http://jid.oxfordjournals.org/ at University of Rochester on July 27, 2015

indicated a perception that serum biomarkers coupled with viral testing would be most helpful to guide antibiotic decisions. Nevertheless, confidence in PCT-guided algorithms among US physicians will likely be an iterative process. Moreover, because early diagnosis and treatment with antibiotics is recommended by professional societies for patients hospitalized with pneumonia and mandated in clinical practice, the potential to prevent inappropriate antibiotic use in the setting of viral LRTI is likely greatest in the population of patients without clinical and diagnostic evidence of a definite pneumonic process. We therefore believe that initial US clinical trials for PCT-guided care of LRTI should focus on persons at lower risk for invasive bacterial disease and without definitive pneumonia. Thus, we investigated the feasibility of conducting PCT-based algorithm trials in a US hospital and the value of concurrent viral testing to reduce unnecessary antibiotic use in adults hospitalized with nonpneumonic LRTI.

METHODS

Study Design

The trial was designed as a 1-year open label randomized clinical trial in which 300 patients hospitalized with non-pneumonic LRTI were randomized 1:1 to standard care or PCT-guided care in combination with multiplex viral PCR testing. The trial was registered on ClinicalTrials.gov (NCT01907659).

Site

Rochester General Hospital (RGH), a 528-bed community hospital in Rochester, New York, was the study site. RGH uses an electronic medical record (EMR), and most inpatients are cared for by staff of the Department of Internal Medicine. A duplex PCR for influenza virus and respiratory syncytial virus (RSV; hereafter, "hospital PCR") is routinely available, and PCT testing is only available for patients in intensive care units (ICUs). RGH was also the site of a previous respiratory illness surveillance study (conducted during 2008–2011), for which the study population and inclusion criteria were identical to those for the present study [15].

Information Sessions

Prior to the study, physicians and midlevel providers (nurse practitioners and physician assistants) were formally educated regarding results of the previous surveillance study, causes of respiratory infections, antibiotic guidelines and antibiotic complications, and the use of PCT algorithms to guide antibiotic therapy.

Subject Recruitment

Adults ≥21 years of age with symptoms compatible with LRTI (ie, admission diagnosis of pneumonia, acute exacerbations of chronic obstructive pulmonary disease [COPD], bronchitis, asthma, influenza, viral syndrome, respiratory failure, and congestive heart failure [CHF]) were identified by reviewing the daily admission census. Patients with characteristics indicative of a high risk for bacterial infection (ie, ICU requirement, active chemotherapy or radiation, immunosuppression, definitive infiltrate on chest radiograph, enrollment systolic blood pressure of <90 mm Hg, and ≥15% band forms in peripheral blood) were excluded. Infiltrates were considered definitive if they were characterized as "unequivocal" on a radiology report. Persons with a clinical diagnosis of pneumonia on admission but ambiguous chest radiograph findings, such as a "possible infiltrate" or "infiltrate versus atelectasis," were not excluded. Patients who had conditions known to increase PCT levels (ie, trauma, renal failure, and pancreatitis) or who received antibiotics prior to admission were excluded. Subjects or their healthcare representative provided written informed consent and the study approved by the RGH and University of Rochester institutional review boards.

Enrollment Procedures

Enrollment was conducted in the morning within 24 hours after admission. At enrollment, demographic, clinical, and laboratory information and nose and throat swab specimens for PCR were collected. Serum samples were collected at admission and at least 12 hours later for PCT testing. Subjects were stratified by the presence of COPD and were randomly assigned at a ratio of 1:1, using blocks of 4, to receive standard care or the intervention. Notation was placed in the EMR for all subjects to indicate their study participation. Subjects randomly assigned to the intervention group had PCT and viral testing performed immediately. Subjects in the standard care group had samples frozen and tested at study termination.

Standard Care

Standard of care testing (bacterial and viral cultures of respiratory samples, hospital influenza/RSV duplex PCR [hospital PCR], and urine legionella antigen analysis) were obtained at the discretion of the providing team. The turnaround time for the hospital PCR was generally 1–2 hours after the sample was received. Urine pneumococcal antigen was not available in the hospital during the study period. Antibiotic decisions were made by the attending physician without intervention by investigators.

Intervention

All standard of care diagnostic tests (bacterial and viral cultures of respiratory samples, hospital PCR, and urine legionella antigen analyses) were ordered at the discretion of the care-providing team. Serum PCT and viral/atypical pathogen PCR testing were performed as soon as possible. Two serum PCT levels were obtained, and the higher of the 2 was used for algorithm interpretation and data analysis. Results were reported in the EMR 2–3 hours after enrollment, and the treating team

Downloaded from http://jid.oxfordjournals.org/ at University of Rochester on July 27, 2015

notified by a text sent via page and by a simultaneous e-mail providing the PCT algorithm. The algorithm was also available on the hospital website and previously distributed pocket cards. The following information was communicated: "Serum PCT is a biomarker associated with bacterial infection, and can be used to guide therapy according to the highest value on admission OR at 12–24 hours. However, PCT is not a substitute for clinical judgment. For PCT values of ≤0.1 ng/mL, initiation of antibiotic treatment is strongly discouraged; for values of 0.11–0.24 ng/mL, initiation is discouraged; for values of 0.25–0.49 ng/mL, initiation is encouraged; and for values of ≥0.5 ng/mL, initiation is strongly encouraged."

Hospital Course Evaluation and Illness Follow-up

Study personnel reviewed the EMR daily until discharge, with attention to antibiotic use and safety outcomes (progression or new pneumonia, lung abscess, empyema, ICU care, respiratory failure, and death). A serious adverse event (SAE) was defined as ICU transfer or death within 30 days of enrollment. All SAEs were reviewed by an independent data safety monitoring board. Subjects were contacted by phone at 30 days and 3 months by personnel blinded to randomization, who collected information about healthcare utilization, antibiotic use and complications, and return to baseline health.

Laboratory Methods

Serum PCT Level

Serum PCT levels were measured by VIDAS BRAHMS (bioMerieux), using the enzyme-linked fluorescent assay technique. The assay range is 0.05–200 ng/mL.

Multiplex PCR

Nose and throat swab specimens were tested using the Film Array Respiratory Panel (FilmArray; Idaho Technologies, Salt Lake City, Utah), which detects 14 common respiratory viruses (adenovirus, coronavirus 229E, coronavirus HKU1, coronavirus NL63, coronavirus OC43, human metapneumovirus, human rhinovirus/enterovirus, influenza A virus, influenza B virus, parainfluenza virus 1, parainfluenza virus 2, parainfluenza virus 3, parainfluenza virus 4, and respiratory syncytial virus) and 3 atypical bacteria (*Bordetella pertussis, Chlamydophila pneumoniae*, and *Mycoplasma pneumoniae*).

End Points

The feasibility end point for the trial was the ability to recruit and randomly assign 300 subjects in a 12-month period. The primary impact end point was duration of antibiotic therapy. A day of antibiotic therapy was defined as any day in which any doses of antibiotics were administered. Other measures of antibiotic exposure included discontinuation of antibiotics within 48 hours and discharge receiving antibiotics. Seven subjects discharged receiving long-term antibiotic therapy for anti-inflammatory properties were excluded from the analyses comparing the number of subjects discharged receiving antibiotics. Safety was assessed during the hospital stay, at 1 and 3 months. Safety end points included length of hospital stay and, 1 and 3 months after discharge, respiratory complications, ICU care, death, and healthcare utilization.

Statistical Analysis

Sample size was chosen to ensure the feasibility of a 1-year pilot study, and therefore formal sample size and power calculations were not performed. Categorical variables were summarized by counts and proportions and compared using the Fisher exact test. Medians and interquartile ranges (IQRs) were used to describe continuous variables, with comparisons performed using the nonparametric Wilcoxon test. For intervention patients with a PCT level of; ≤0.24 ng/mL, logistic regression was used to model algorithm compliance as a function of clinical covariates, including age, length of symptoms prior to admission, sputum culture results, signs and symptoms of illness, admission diagnosis, chest radiograph results, PCT level, and viral testing. SAS 9.4 was used for all analyses, with tests performed at the 2-sided 0.05 level.

RESULTS

Subject and Illness Characteristics

The planned sample size of 300 subjects was achieved in 7 months. From October 2013 through April 2014, 685 hospitalized patients were assessed; 151 eligible patients were randomly assigned to the intervention group, and 149 were randomly assigned to the nonintervention group (Figure 1). The 2 groups were well matched for demographic characteristics, underlying medical conditions, admission diagnoses, and severity of illness, with the exception that a significantly greater percentage patients with CHF was randomly assigned to the nonintervention group (Table 1). The most common admission diagnoses in both groups were acute exacerbations of COPD (38%–39%), asthma exacerbation (18%–21%), pneumonia (19%), CHF (6%–11%), and influenza (6%–7%).

Laboratory Data

Overall, 132 viral diagnoses (64 in the intervention group and 64 in the nonintervention group) were made by any means in 128 subjects (4 subjects had 2 distinct viruses). FilmArray testing was performed for all intervention subjects during the study period and for all nonintervention subjects after study completion. In the intervention group, hospital PCR detected influenza virus or RSV in 13%, whereas the FilmArray test detected viral RNA in 42%. Notably, 19% of nonintervention subjects had positive results of hospital PCR, which was available to treating physicians; the most common viruses detected were influenza A virus (in 33% of cases), rhinovirus (19%), RSV (17%), human coronavirus (14%), and human metapneumovirus (8%).

Downloaded from http://jid.oxfordjournals.org/ at University of Rochester on July 27, 2015

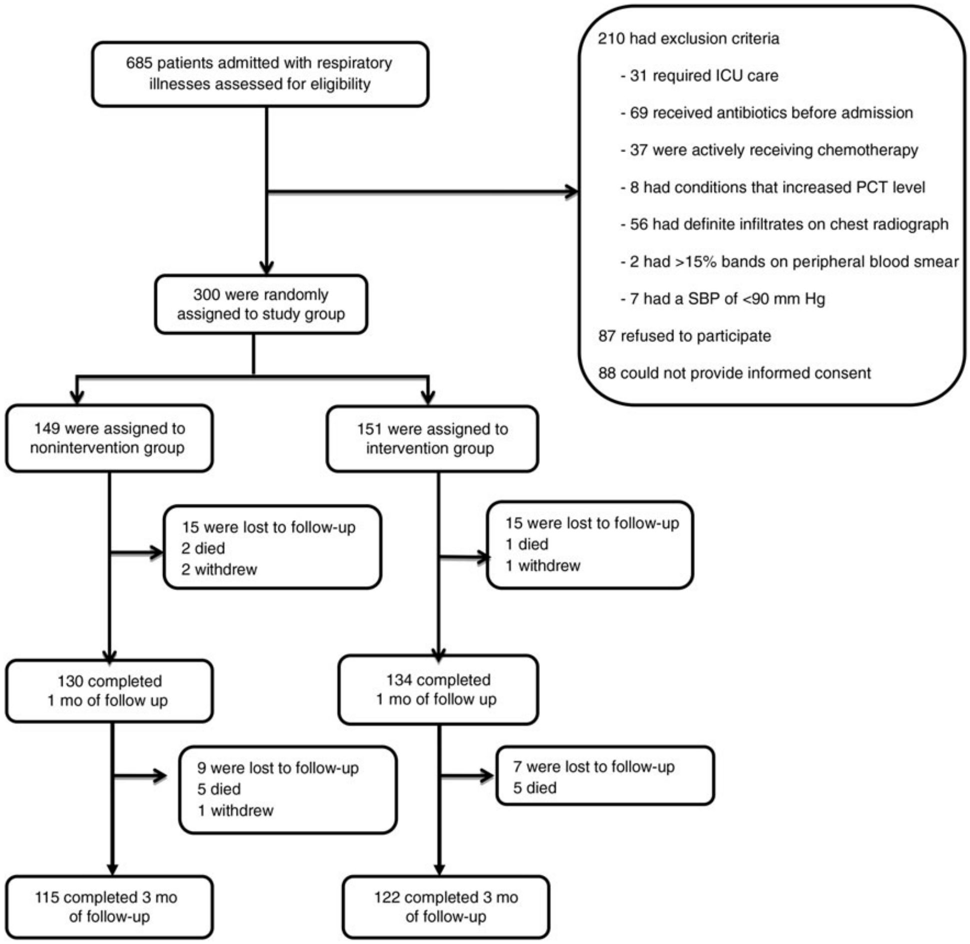

Figure 1. Flow of patients through the study. Reasons for exclusion from the study included intensive care unit (ICU) stay, antibiotic use for >24 hours prior to enrollment, active chemotherapy, conditions known to increase procalcitonin level (eg, renal failure, pancreatitis, and trauma), definite infiltrate on a chest radiograph (according to radiology report), >15% bands on a peripheral blood smear, and a systolic blood pressure (SBP) of <90 mm Hg at enrollment. Abbreviation: PCT, procalcitonin.

The majority of patients in both groups had low admission PCT values (≤0.24 ng/mL; Table 1). All 151 intervention patients had data on their serum PCT level at admission, and 139 had data from a subsequent measurement performed on day 2 of hospitalization. Of the 126 intervention subjects with an initial low PCT value, 5 had a higher second level, resulting in 121 intervention subjects with PCT values of ≤0.24 ng/mL at both time points.

Bacterial diagnoses were made among 9%–10% of patients, with most made on the basis of sputum culture results and 25% associated with high PCT levels (>0.24 ng/mL). Three patients had positive results of a FilmArray assay for atypical bacteria, and illnesses in all 3 were associated with low serum PCT levels. There were 216 bacterial blood cultures performed for subjects in both arms, and all but 1 had negative results. The exception involved 1 nonintervention patient, who had *Streptococcus pneumoniae* bacteremia. This illness was associated with a high serum PCT level on admission.

Primary Outcomes

Antibiotic exposure was measured on the basis of discontinuation of antibiotic treatment within 48 hours, discharge receiving oral antibiotics, and total duration of therapy (Table 2). There were no significant differences in duration of antibiotic therapy between intervention and nonintervention patients, although there was a trend toward a decreased number of intervention patients discharged receiving antibiotics (35% vs 44%; $P = .09$; Table 2*A*).

When antibiotic exposure in the intervention subgroup with presumably the lowest risk for bacterial infection (ie, patients who tested positive for virus and had a low PCT level) was compared to that in the nonintervention group, we noted a trend toward fewer days of antibiotics prescribed (median, 2 days [IQR, 1–6 days] vs 4 days [IQR, 0–8 days]; $P = .11$), with significantly fewer patients discharged receiving antibiotics (20% vs 45%; $P = .002$; Table 2*B* and Figure 2). Analysis of antibiotic use among subjects for whom treating physicians adhered to

Downloaded from http://jid.oxfordjournals.org/ at University of Rochester on July 27, 2015

Table 1. Patient Characteristics

Characteristic	Intervention Group (n = 151)	Nonintervention Group (n = 149)	*P* Value
Age, y	61 (51–72)	64 (50–74)	.41
Female sex	88 (58)	80 (54)	.49
Race/ethnicity			
Black	53 (35)	37 (25)	.06
White	98 (65)	111 (75)	.08
Hispanic	12 (8)	16 (11)	.43
Underlying condition			
Current/former smoker	123 (81)	126 (85)	.54
Diabetes mellitus	60 (40)	48 (32)	.19
COPD	72 (48)	66 (44)	.56
CHF	33 (22)	50 (34)	.03
Asthma	47 (31)	41 (28)	.53
Hypertension	88 (58)	92 (62)	.56
Sign/symptom			
Fever[a]	32 (21)	30 (20)	.88
Cough	138 (91)	134 (90)	.70
Wheeze	125 (83)	126 (85)	.76
Dyspnea	140 (93)	136 (91)	.68
Sputum production	118 (78)	92 (62)	.002
Admission diagnosis			
Pneumonia	28 (19)	29 (19)	.88
Influenza	9 (6)	11 (7)	.65
CHF	9 (6)	16 (11)	.15
COPD	58 (39)	58 (38)	.99
Asthma	32 (21)	27 (18)	.56
Laboratory finding			
WBC count, $\times 10^3$ cells/µL	9.1 (6.5–11.5)	9.3 (7.3–12.1)	.50
CURB-65 score	1.0 (0.0–2.0)	2.0 (1.0–2.0)	.16
Chest radiography finding[b]			
No acute disease	72 (49)	87 (58)	.10
Possible infiltrate	41 (28)	26 (17)	.03
Viral diagnosis			
Test type			
FilmArray PCR[c]	63 (42)	64 (40)	.99
Hospital PCR[d]	20 (13)	28 (19)	.26
PCR-positive result	64 (42)	64 (43)	
Bacterial diagnosis			
Test type			
PCR	2 (1)	1 (1)	.99
Culture	13 (9)	12 (8)	.99
Admission PCT level, ng/mL[c,e]	0.05 (0.05–0.11)	0.05 (0.05–0.11)	.65
≤0.10	112 (74)	107 (74)	.99
0.11–0.24	14 (9)	19 (13)	.36
0.25–0.50	10 (7)	10 (7)	.99
≥0.50	15 (10)	9 (6)	.29

Data are no. (%) of patients or median value (interquartile range). See "Methods" section for descriptions of the intervention and nonintervention groups.

Abbreviations: CHF, congestive heart failure; COPD, chronic obstructive pulmonary disease; PCR, polymerase chain reaction; PCT, procalcitonin; WBC, white blood cell.

[a] Defined as a temperature of ≥38°C.

[b] Three intervention patients did not undergo chest radiography.

[c] Data for nonintervention patients were not available to clinicians during the trial.

[d] Defined as a duplex PCR for influenza virus and respiratory syncytial virus.

[e] Four nonintervention patients did not have the PCT level measured.

the algorithm revealed a significantly shorter duration of therapy, compared with the duration among nonintervention subjects (median, 2 days [IQR, 0–3 days] vs 4 days [IQR, 0–8 days]; $P = .004$; Table 2*C*).

Consistent with these findings, intervention subjects with low PCT values were less likely to receive antibiotics for ≥48 hours or at discharge than those with high PCT levels (Table 3*A*). The total duration of antibiotic therapy were also shorter for subjects with low PCT levels (median, 2 days [IQR, 0–6 days] vs 7.5 days [IQR, 5–10 days]; $P < .001$; Table 3*A*). However, patients with high PCT values also had higher CURB-65 scores (median, 2 [IQR, 1–3] vs 1 [IQR, 0–2]; $P = .001$), and a greater percentage showed possible infiltrates on chest radiograph (40% vs 25%; $P = .12$). Additional subgroup analyses assessing the added value of viral testing to PCT algorithms were performed. Patients with a known viral diagnosis during hospitalization (64 in the intervention group and 28 in the nonintervention group) were less frequently discharged receiving antibiotics (28% vs 45%; $P = .01$), with a trend toward a shorter duration of therapy (median, 2 days [IQR, 1–6 days] vs 4 days [IQR, 1–8 days]; $P = .07$; Table 3*B*), compared with those without a viral diagnosis.

We also investigated the possibility of a study effect on the nonintervention arm resulting from prestudy educational sessions and from subjects in both arms receiving care from the same providers. Primary outcomes for the nonintervention group were compared to those for matched historical control subjects who were hospitalized at RGH with LRTI during a 2008–2011 surveillance study and were deemed, using the identical criteria, to be at low risk for bacterial infection [15]. There were no significant differences in demographic characteristics, admission diagnoses, and illness severity between the groups. However, compared with historical controls, there were significant decreases in antibiotic exposure in the nonintervention group, most notably a 2-day

Downloaded from http://jid.oxfordjournals.org/ at University of Rochester on July 27, 2015

Table 2. Comparison of Antibiotic Use Between the Intervention Group/Subgroups or Historical Controls and the Nonintervention Group

Characteristic	Intervention Group	Nonintervention Group	*P* Value
Subjects, no.	151	149	
Antibiotic use for ≤48 h	69 (46)	61 (41)	.42
Discharged receiving oral antibiotics	51 (35)[a]	64 (44)[b]	.09
Total antibiotic-days	3.0 (1.0–7.0)	4.0 (0.0–8.0)	.71
	Intervention Subgroup Positive for Virus With Low PCT Values	Nonintervention Group	
Subjects, no.	49	149	
Antibiotic use for ≤48 h	28 (57)	61 (41)	.07
Discharged receiving oral antibiotics	10 (20)	64 (45)[b]	.002
Total antibiotic-days	2.0 (1.0–6.0)	4.0 (0.0–8.0)	.11
	Intervention Subgroup Adherent to Algorithm	Nonintervention Group	
Subjects, no.	96	149	
Antibiotic use for ≤48 h	63 (65)	61 (41)	.002
Discharged receiving oral antibiotics	19 (20)[c]	64 (45)[b]	.002
Total antibiotic-days	2.0 (0.0–3.0)	4.0 (0.0–8.0)	.004
	Historical Controls	Nonintervention Group	
Subjects, no.	586	149	
Antibiotic use for ≤48 h	150 (24)	61 (41)	<.001
Discharged receiving oral antibiotics	342 (56)[d]	64 (45)[b]	.003
Total antibiotic-days	6.0 (2.0–9.0)	4.0 (0.0–8.0)	<.001

Data are no. (%) of patients or median no. (interquartile range). See "Methods" section for descriptions of the intervention and nonintervention groups and the historical controls.

Abbreviation: PCT, procalcitonin.

[a] Data are for 147 subjects.

[b] Data are for 144 subjects.

[c] Data are for 93 subjects.

[d] Data are for 616 subjects.

decrease in the duration of antibiotic therapy (median, 6 days [IQR, 2–9 days] vs 4 days [IQR, 0–8 days]; *P* < .001; Table 2*D*).

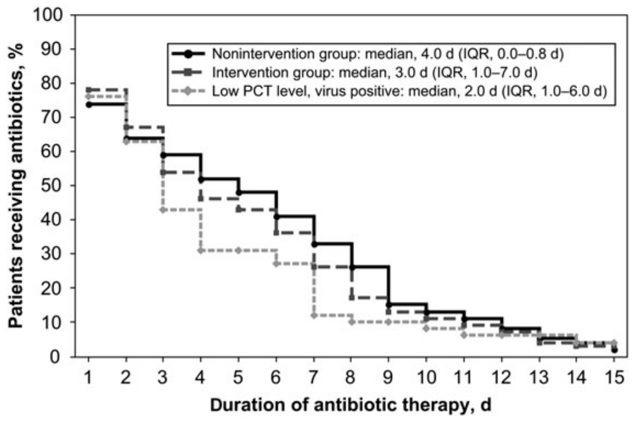

Figure 2. Time series plot of total antibiotic days in intervention and nonintervention patients and a subgroup analysis of virus-positive intervention subjects with low procalcitonin (PCT) levels, compared with the nonintervention arm. Abbreviation: IQR, interquartile range.

Secondary Outcomes

Safety was assessed during the hospital stay, at 1 and 3 months. No deaths occurred during hospitalization, and 4 SAEs occurred in each arm of the study, with none judged to be related to the intervention. One intervention patient and 4 nonintervention patients developed a new cases of pneumonia within 30 days. The median length of hospital stay was 4 days in both groups, and the number of posthospitalization healthcare visits was similar. Of note, the length of illness (assessed from the onset of symptoms prior to hospitalization to the subject's report of a return to baseline) was shorter in intervention patients (median, 16 days [IQR, 12–24 days] vs 20 days [IQR, 13–28 days]; *P* = .03). There were 27 potential antibiotic adverse events (rash, gastrointestinal symptoms, fungal overgrowth, or resistant flora) in the intervention arm and 28 in the nonintervention arm. Three cases of *Clostridium difficile* colitis occurred in nonintervention patients, compared with none in the intervention group. At 3 months, there were no significant differences in any of the clinical safety outcomes for the intervention arm or the subgroup of algorithm-adherent intervention subjects, compared with nonintervention patients.

Downloaded from http://jid.oxfordjournals.org/ at University of Rochester on July 27, 2015

Table 3. Comparison of Antibiotic Use in the Intervention Group, by Procalcitonin (PCT) Level, and Among All Patients, by Results of Viral Testing

Characteristic	Low PCT Level	High PCT Level	*P* Value
Subjects, no.	121	30	
Antibiotic use for ≤48 h	63 (52)	6 (20)	.002
Discharged receiving oral antibiotics	32 (27)[a]	19 (63)	<.001
Total antibiotic-days	2.0 (0.0–6.0)	7.5 (5.0–10.0)	<.001
	Virus Positive	Virus Negative	
Subjects, no.	92	208	
Antibiotic use for ≤48 h	47 (51)	83 (40)	.08
Discharged receiving oral antibiotics	26 (28)	89 (45)[b]	.01
Total antibiotic-days	2.0 (1.0–6.0)	4.0 (1.0–8.0)	.07

Data are no. (%) of patients or median no. (interquartile range). See "Methods" section for descriptions of the intervention and nonintervention groups.

[a] Data are for 117 subjects.

[b] Data are for 198 subjects.

Healthcare Provider Adherence to the Algorithm

Overall, algorithm adherence was 64%, although providers were more likely to prescribe antibiotics for patients with high PCT values than to withhold antibiotics for patients with low PCT values (Figure 3). The majority of providers (77%) followed algorithm recommendations to continue antibiotic treatment when PCT values were high, although it was notable that antibiotic therapy was discontinued in 5 subjects with high PCT values. In contrast, providers followed algorithm recommendations to discontinue antibiotics in 61% of subjects for whom a low PCT value was detected. The only variable associated with algorithm nonadherence in subjects with low PCT values was an admission diagnosis of pneumonia (26% vs 7%; $P = .01$).

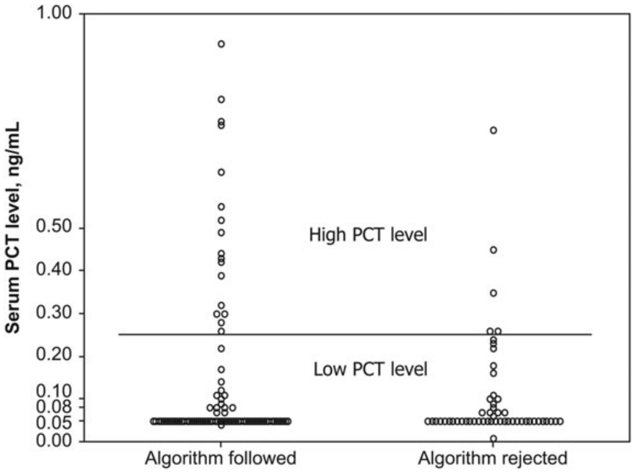

Figure 3. Provider response to the procalcitonin (PCT)–guided treatment algorithm. A circle represents an individual PCT value for each intervention study subject. The horizontal bar represents the threshold for PCT values (0.24 ng/mL), which defines levels as either low or high. The algorithm discourages antibiotic use below this threshold and recommends antibiotics for values above this level. Results are segregated by provider response to the algorithm and are designated as "algorithm followed" and "algorithm rejected."

DISCUSSION

This is the first study combining PCT measurement with molecular viral diagnostic testing, as well as the first randomized clinical trial performed solely in the United States that evaluated PCT-guided care for patients hospitalized with respiratory illnesses. Although overall antibiotic exposure was similar in the intervention and nonintervention arms, subgroup analyses and comparison with historical controls were encouraging because they suggested that US physicians will respond to viral and biomarker data to inform antibiotic use. The primary goal of our study was to evaluate the feasibility of conducting randomized clinical trials of PCT-guided antibiotic recommendations in the United States. Our data clearly indicate that such trials are likely to be well received, since complete enrollment was achieved 5 months early.

Evidence is mounting that PCT-guided treatment of respiratory infections can safely reduce antibiotic use [10, 16–20]. European trials have been performed in a variety of settings, including emergency departments, primary care offices, hospitals, and ICUs [10, 16, 17, 19–25]. In all prior studies, antibiotic use was decreased without harm, as measured by composite adverse event outcomes. The largest trial to date was a Swiss multicenter randomized trial of PCT-guided therapy in 1359 hospitalized patients with LRTI [16]. A 35% decrease in overall antibiotic exposure was reported without notable differences in short-term or long-term outcomes. Currently, use of PCT levels in the United States is only approved for management of sepsis [11, 26]. The only US data on PCT-guided care for patients with LRTI comes from the ProREAL study, an observational

Downloaded from http://jid.oxfordjournals.org/ at University of Rochester on July 27, 2015

surveillance of antibiotic prescribing practices when PCT testing was made available in one US hospital, 10 Swiss centers and 3 French centers [22, 23]. For the 295 patients evaluated in the United States, adherence to the algorithm was 35%, which was significantly lower than the percentage among algorithm-experienced European centers, suggesting that experience increases confidence and compliance with this approach.

In contrast, studies examining the benefits of viral testing to reduce unnecessary antibiotic use have largely been observational [27–32]. The only randomized clinical trial to date was performed in children and demonstrated significantly less antibiotic use and diagnostic testing for children whose rapid influenza test results were made available to providers, compared with those whose information was withheld [33]. Although limited by the small sample size, our study suggests that testing for viral pathogens in addition to influenza virus may reduce antibiotic use.

Because confidence in PCT-guided algorithms among US physicians will likely be an iterative process, we focused on patients with nonpneumonic LRTI who were at low risk for bacterial infection, using standard clinical parameters. Adherence to the PCT algorithm was 64%, which was encouraging, given the low US compliance rate in the study by Albrich et al [22, 23]. However, despite relatively good compliance and a population enriched for subjects with a low risk for bacterial complications, we did not see significant differences in antibiotic use between the intervention and nonintervention arms. We suspect that this result was in part due to a significant study effect resulting in decreased antibiotic exposure in nonintervention patients. Supporting this conclusion was the overall decrease in the duration of antibiotic therapy for nonintervention subjects, compared with the duration for similar historical controls. The study effects were likely multifactorial, including the prestudy educational sessions, spillover care resulting from providers caring for patients in both arms, and increased awareness of viral activity in the community. Last, providers were also aware that their behavior was being observed, which may have resulted in a Hawthorne effect [34].

Our study had a number of limitations. Because the trial was designed as a feasibility pilot with a small sample size, definitive conclusions cannot be drawn from subgroup analyses, particularly the additive value of viral testing to PCT-guided therapy. Moreover, the study was not powered to determine noninferiority of PCT-guided care, compared with standard treatment algorithms. However, like the European trials, we did not detect any major safety signals prohibitive to proceeding with larger clinical trials. Second, repeat PCT measurements over several days during hospitalization were not performed and may be of added value to decrease antibiotic use. Additionally, routinely available hospital PCR may have diluted the effects of multiplex viral testing. Last, the use of historical controls to evaluate a study effect cannot address other changes in practice that may have occurred unrelated to the study.

Nevertheless, the lessons learned from this study should facilitate the design of larger, definitive US trials. Although our study clearly demonstrates the feasibility of performing PCT trials in the United States, a number of factors need consideration. First, hospital stays for nonpneumonic LRTI tend to be brief, and thus the opportunity to intervene is limited. To maximize impact on antibiotic exposure, the intervention should begin early, ideally in the emergency department. Second, algorithm adherence needs to improve, although clinical judgment remains the cornerstone of good medical care, and 100% compliance should not be the goal. Future trials should therefore incorporate strategies to influence physician behavior, such as the use of antibiotic stewardship teams to reinforce PCT algorithm recommendations. Because a study effect appeared to be an important impediment to demonstrating a significant difference in antibiotic exposure or the independent effect of viral testing, future trials may need to use separate but comparable study sites for the intervention and nonintervention groups. Finally, once the safety of PCT-guided care in patients with LRTI at lower risk for bacterial infections has been established, its use should also be explored for subjects with pneumonic disease.

In conclusion, despite the modest changes in antibiotic exposure observed in the primary analysis, the results of the subgroup analyses and comparison with historical controls are encouraging because they suggest that US physicians will respond to viral and biomarker data. Ultimately, further studies are needed to assess the value of viral testing and to determine the role of PCT-guided care as a practical and effective treatment protocol for patients admitted with nonpneumonic respiratory illnesses.

Notes

Acknowledgments. We thank the patients and Rochester General Hospital staff, particularly the members of the clinical microbiology and specimen receiving departments, for their help in conducting this study; Jami McGrath, for sample processing; Patricia Hennessey, for data collection; and the members of our data safety monitoring board, Andrew J. Swinburne, MD, Alexandra Yamshchikov, MD, and Walter Poleshenski, MD. None of the listed received any financial compensation for their contributions.

Financial support. This work was supported by Rochester General Hospital (KIDD Fund); the National Institutes of Health, National Institute of Allergy and Infectious Diseases (contract HHSN27220120005C); and BioFire (FilmArray respiratory panel instrument).

Potential conflicts of interest. A. R. F. has served on advisory boards for Novavax, Hologic, Gilead, and Medimmune and has received research funding from AstraZeneca, Sanofi Pasteur, GlaxoSmithKline, and ADMA Biologics. E. E. W. has served on advisory boards for Novavax, Gilead, Alios Pharmaceuticals, ClearPath, and Medimmune and has received research funding from AstraZeneca and Sanofi Pasteur. All other authors report no potential conflicts.

All authors have submitted the ICMJE Form for Disclosure of Potential Conflicts of Interest. Conflicts that the editors consider relevant to the content of the manuscript have been disclosed.

References

1. Widmer K, Zhu Y, Williams JV, Griffin MR, Edwards KM, Talbot HK. Rates of hospitalizations for respiratory syncytial virus, human

Downloaded from http://jid.oxfordjournals.org/ at University of Rochester on July 27, 2015

metapneumovirus, and influenza virus in older adults. J Infect Dis **2012**; 206:56–62.
2. Falsey AR, Hennessey PA, Formica MA, Cox C, Walsh EE. Respiratory syncytial virus infection in elderly and high-risk adults. N Engl J Med **2005**; 352:1749–59.
3. Dowell SF, Anderson LJ, Gary HE Jr, et al. Respiratory syncytial virus is an important cause of community-acquired lower respiratory infection among hospitalized adults. J Infect Dis **1996**; 174:456–62.
4. Cameron RJ, de Wit D, Welsh TN, Ferguson J, Grissell TV, Rye PJ. Virus infection in exacerbations of chronic obstructive pulmonary disease requiring ventilation. Intensive Care Med **2006**; 32:1022–9.
5. Templeton KE, Scheltinga SA, van den Eeden WC, Graffelman AW, van den Broek PJ, Claas EC. Improved diagnosis of the etiology of community-acquired pneumonia with real-time polymerase chain reaction. Clin Infect Dis **2005**; 41:345–51.
6. Oosterheert JJ, van Loon AM, Schuurman R, et al. Impact of rapid detection of viral and atypical bacterial pathogens by real-time polymerase chain reaction for patients with lower respiratory tract infection. Clin Infect Dis **2005**; 41:1438–44.
7. Peltola VT, McCullers JA. Respiratory viruses predisposing to bacterial infections: role of neuraminidase. Pediatr Infect Dis J **2004**; 23:S87–97.
8. Paterson DL. "Collateral damage" from cephalosporin or quinolone antibiotic therapy. Clin Infect Dis **2004**; 38(suppl 4):S341–5.
9. Muto CA, Pokrywka M, Shutt K, et al. A large outbreak of *Clostridium difficile*-associated disease with an unexpected proportion of deaths and colectomies at a teaching hospital following increased fluoroquinolone use. Infect Control Hosp Epidemiol **2005**; 26:273–80.
10. Schuetz P, Chiappa V, Briel M, Greenwald JL. Procalcitonin algorithms for antibiotic therapy decisions: a systematic review of randomized controlled trials and recommendations for clinical algorithms. Arch Intern Med **2011**; 171:1322–31.
11. Becker KL, Snider R, Nylen ES. Procalcitonin assay in systemic inflammation, infection, and sepsis: clinical utility and limitations. Crit Care Med **2008**; 36:941–52.
12. Gilbert DN. Procalcitonin as a Biomarker in Respiratory Tract Infection. Clin Infect Dis **2011**; 52:S362.
13. Martinez FJ, Curtis JL. Procalcitonin-guided antibiotic therapy in COPD exacerbations: closer but not quite there. Chest **2007**; 131:1–2.
14. FDA-IDSA public workshop: advancing clinical development of molecular and other diagnostic tests for respiratory tract infections. 12–13 November 2009. http://www.gpo.gov/fdsys/pkg/FR-2009-10-15/html/E9-24828.htm. Accessed 16 May 2001.
15. Falsey AR, Becker KL, Swinburne AJ, et al. Bacterial complications of respiratory tract viral illness: a comprehensive evaluation. J Infect Dis **2013**; 208:432–41.
16. Schuetz P, Christ-Crain M, Thomann R, et al. Effect of procalcitonin-based guidelines vs standard guidelines on antibiotic use in lower respiratory tract infections: the ProHOSP randomized controlled trial. JAMA **2009**; 302:1059–66.
17. Briel M, Schuetz P, Mueller B, et al. Procalcitonin-guided antibiotic use vs a standard approach for acute respiratory tract infections in primary care. Arch Intern Med **2008**; 168:2000–7; discussion 2007–8.
18. Briel M, Christ-Crain M, Young J, et al. Procalcitonin-guided antibiotic use versus a standard approach for acute respiratory tract infections in primary care: study protocol for a randomised controlled trial and baseline characteristics of participating general practitioners [ISRCTN73182671]. BMC Fam Pract **2005**; 6:34.
19. Christ-Crain M, Stolz D, Bingisser R, et al. Procalcitonin guidance of antibiotic therapy in community-acquired pneumonia: a randomized trial. Am J Respir Crit Care Med **2006**; 174:84–93.
20. Christ-Crain M, Jaccard-Stolz D, Bingisser R, et al. Effect of procalcitonin-guided treatment on antibiotic use and outcome in lower respiratory tract infections: cluster-randomised, single-blinded intervention trial. Lancet **2004**; 363:600–7.
21. Muller F, Christ-Crain M, Bregenzer T, et al. Procalcitonin levels predict bacteremia in patients with community-acquired pneumonia: a prospective cohort trial. Chest **2010**; 138:121–9.
22. Albrich WC, Dusemund F, Bucher B, et al. Effectiveness and safety of procalcitonin-guided antibiotic therapy in lower respiratory tract infections in "real life": an international, multicenter poststudy survey (ProREAL). Arch Intern Med **2012**; 172:715–22.
23. Dusemund F, Bucher B, Meyer S, et al. Influence of procalcitonin on decision to start antibiotic treatment in patients with a lower respiratory tract infection: insight from the observational multicentric ProREAL surveillance. Eur J Clin Microbiol Infect Dis **2013**; 32:51–60.
24. Tang J, Long W, Yan L, et al. Procalcitonin guided antibiotic therapy of acute exacerbations of asthma: a randomized controlled trial. BMC Infect Dis **2013**; 13:596.
25. Stolz D, Christ-Crain M, Bingisser R, et al. Antibiotic treatment of exacerbations of COPD: a randomized, controlled trial comparing procalcitonin-guidance with standard therapy. Chest **2007**; 131:9–19.
26. Simon L, Gauvin F, Amre DK, Saint-Louis P, Lacroix J. Serum procalcitonin and C-reactive protein levels as markers of bacterial infection: a systematic review and meta-analysis. Clin Infect Dis **2004**; 39:206–17.
27. Shiley KT, Lautenbach E, Lee I. The use of antimicrobial agents after diagnosis of viral respiratory tract infections in hospitalized adults: antibiotics or anxiolytics? Infect Control Hosp Epidemiol **2010**; 31:1177–83.
28. Esposito S, Marchisio P, Morelli P, Crovari P, Principi N. Effect of a rapid influenza diagnosis. Arch Dis Childhood **2003**; 88:525–6.
29. Poehling KA, Zhu Y, Tang Y, Edwards K. Accuracy and impact of a point-of-care rapid influenza test in young children with respiratory illness. Arch Pediatr Adolesc Med **2006**; 160:713–8.
30. Sharma V, Dowd MD, Slaughter AJ, Simon SD. Effect of rapid diagnosis of influenza virus type A on the emergency department management of febrile infants and toddlers. Arch Pediatr Adolesc Med **2002**; 156:41–3.
31. Noyola DE, Rodriguez-Moreno G, Sanchez-Alvarado J, Martinez-Wagner R, Ochoa-Zavala JR. Viral etiology of lower respiratory tract infections in hospitalized children in Mexico. Pediatr Infect Dis J **2004**; 23:118–23.
32. Falsey AR, Murata Y, Walsh EE. Impact of rapid diagnosis on management of adults hospitalized with influenza. Arch Intern Med **2007**; 167:354–60.
33. Bonner AB, Monroe KW, Talley L, Klasner AE, Kimberlin DW. Impact of the rapid diagnosis of influenza on physician decision-making and patient management in the pediatric emergency department: Results of a randomized, prospective, controlled trial. Pediatrics **2003**; 112:363–7.
34. Claus CK. B. F. Skinner and T. N. Whitehead: A brief encounter, research similarities, Hawthorne revisited, what next? Behav Anal **2007**; 30:79–86.

Downloaded from http://jid.oxfordjournals.org/ at University of Rochester on July 27, 2015
